# Surgical parameters influence paediatric knee kinematics and cartilage stresses in anterior cruciate ligament reconstruction: Navigating subject‐specific variability using neuromusculoskeletal‐finite element modelling analysis

**DOI:** 10.1002/ksa.12413

**Published:** 2024-08-06

**Authors:** Ayda Karimi Dastgerdi, Amir Esrafilian, Christopher P. Carty, Azadeh Nasseri, Martina Barzan, Rami K. Korhonen, Ivan Astori, Wayne Hall, David John Saxby

**Affiliations:** ^1^ Griffith Centre of Biomedical and Rehabilitation Engineering (GCORE) Griffith University Gold Coast Queensland Australia; ^2^ Department of Technical Physics University of Eastern Finland Kuopio Finland; ^3^ Department of Orthopedics Children's Health Queensland Hospital and Health Service Brisbane Queensland Australia; ^4^ School of Engineering and Built Environment, Mechanical Engineering and Industrial Design Griffith University Gold Coast Queensland Australia

**Keywords:** biomechanics, cartilage, computational modelling, gait, in silico, knee, precision medicine, surgical planning

## Abstract

**Purpose:**

Anterior cruciate ligament (ACL) rupture is increasingly common in paediatric and adolescent populations, typically requiring surgical ACL reconstruction (ACLR) to restore knee stability. However, ACLR substantially alters knee biomechanics (e.g., motion and tissue mechanics) placing the patient at elevated risk of early‐onset knee osteoarthritis.

**Methods:**

This study employed a linked neuromusculoskeletal (NMSK)‐finite element (FE) model to determine effects of four critical ACLR surgical parameters (graft type, size, location and pre‐tension) on tibial articular cartilage stresses in three paediatric knees of different sizes during walking. Optimal surgical combinations were defined by minimal kinematic and tibial cartilage stress deviations in comparison to a corresponding intact healthy knee, with substantial deviations defined by normalized root mean square error (nRMSE) > 10%.

**Results:**

Results showed unique trends of principal stress deviations across knee sizes with small knee showing least deviation from intact knee, followed by large‐ and medium‐sized knees. The nRMSE values for cartilage stresses displayed notable variability across different knees. Surgical combination yielding the highest nRMSE in comparison to the one with lowest nRMSE resulted in an increase of maximum principal stress on the medial tibial cartilage by 18.0%, 6.0% and 1.2% for small, medium and large knees, respectively. Similarly, there was an increase of maximum principal stress on lateral tibial cartilage by 11.2%, 4.1% and 12.7% for small, medium and large knees, respectively. Knee phenotype and NMSK factors contributed to deviations in knee kinematics and tibial cartilage stresses. Although optimal surgical configurations were found for each knee size, no generalizable trends emerged emphasizing the subject‐specific nature of the knee and neuromuscular system.

**Conclusion:**

Study findings underscore subject‐specific complexities in ACLR biomechanics, necessitating personalized surgical planning for effective restoration of native motion and tissue mechanics. Future research should expand investigations to include a broader spectrum of subject‐specific factors to advance personalized surgical planning.

**Level of Evidence:**

Level III.

**Abbreviations**
ACL: anterior cruciate ligament
ACLR: anterior cruciate ligament reconstruction
CEINMS: Calibrated EMG‐Informed Neuromusculoskeletal Modelling
DoF: degree of freedom
EMG: electromyography
FE: finite element
ID: inverse dynamics
IK: inverse kinematics
MRI: magnetic resonance imaging
MTU: muscle‐tendon units
NMSK: neuromusculoskeletal
nRMSE: normalized root mean square error
OA: osteoarthritis
TFJ: tibiofemoral joint

## INTRODUCTION

Anterior cruciate ligament (ACL) rupture is an increasingly common injury amongst physically active children and adolescents that often occurs during sports participation [13, 19, 29, 31]. The ruptured ACL is typically reconstructed (ACLR) by surgically implanting a graft (e.g., from gracilis, semitendinosus, or patellar tendon) to replace the failed native ligament with the aim of restoring passive stability to the knee [18]. Despite ACLR, knee laxity often deviates from normal, and ambulatory knee motions, loads, and muscle activation patterns are chronically disrupted [5] which affects knee cartilage mechanics [4, 20, 23, 32]. These altered knee biomechanics may contribute to the development of early onset knee osteoarthritis (OA), which is highly prevalent in ACLR knees one to two decades following injury [4, 20]. The exact mechanisms remain incompletely understood, but impaired knee kinematics and cartilage mechanics (e.g., stress) post‐ACLR are thought influential in subsequent early onset of knee OA [1, 7, 9]. To maximize function and minimize the risk of OA in ACLR knees, optimizing surgical parameters becomes crucial, with primary objective being restoration of normal kinematics and cartilage mechanics to the knee [4, 16, 20, 23].

During ACLR, the surgeon must select graft type, size, pre‐tension and femoral tunnel location amongst other parameters. Each of these surgical parameters in isolation can mechanistically influence the biomechanics of the ACLR knee [3]. In paediatric ACLR literature, no study has systematically evaluated effects of these surgical parameters on knee biomechanics. Further, these surgical parameters likely interact to create complex and non‐intuitive effects on ACLR knee kinematics, kinetics, and cartilage mechanics [3, 16, 20, 23]. Biomechanical outcomes of ACLR are likely also influenced by subject‐specific anatomy (e.g., knee size) and neuromusculoskeletal (NMSK) factors (e.g., motion and loading). Knee anatomy, particularly size, might play a pivotal role, with smaller or larger knee joints exhibiting distinct responses to ACLR [8]. Likewise, subject‐specific NMSK control of knee motion and loading adds further complexity to understanding post‐ACLR biomechanics. Recognizing this complexity, surgical parameters may need to be specific to each patient to ensure the post‐ACLR knee behaves similarly to the intact knee [20, 23]. However, to do so, a physics‐based platform is required to study such a complex mechanical system.

Finite element (FE) models enable study of the effects of ACLR surgical parameters on knee kinematics, kinetics, and tissue‐level mechanics [3, 30], and provides a computational platform to assess surgical optimality. The primary aim of this study was to use a linked NMSK‐FE model to determine the effects of four key surgical parameters (i.e., graft type, size, location and pre‐tension) on knee motions and tibial articular cartilage stresses during walking gait. The secondary aim was to assess if and how subject‐specific variation in knee joint geometry and motion/loading conditions affected tibial articular cartilage stresses. The tertiary aim was to find optimal surgical combinations and examine whether trends emerge in the surgical parameters that produced optimal ACLR knee biomechanics. It was hypothesized that ACLR surgical parameters (i.e., graft type, size, location and pre‐tension) would affect knee motions and tibial cartilage stresses during walking. It was further hypothesized that the response of tibial cartilage stresses to surgical parameters would be affected by subject‐specific knee anatomy (e.g., size) and NMSK control (e.g., motion, loading).

## MATERIALS AND METHODS

### Overview of the workflow

**Figure 1:**
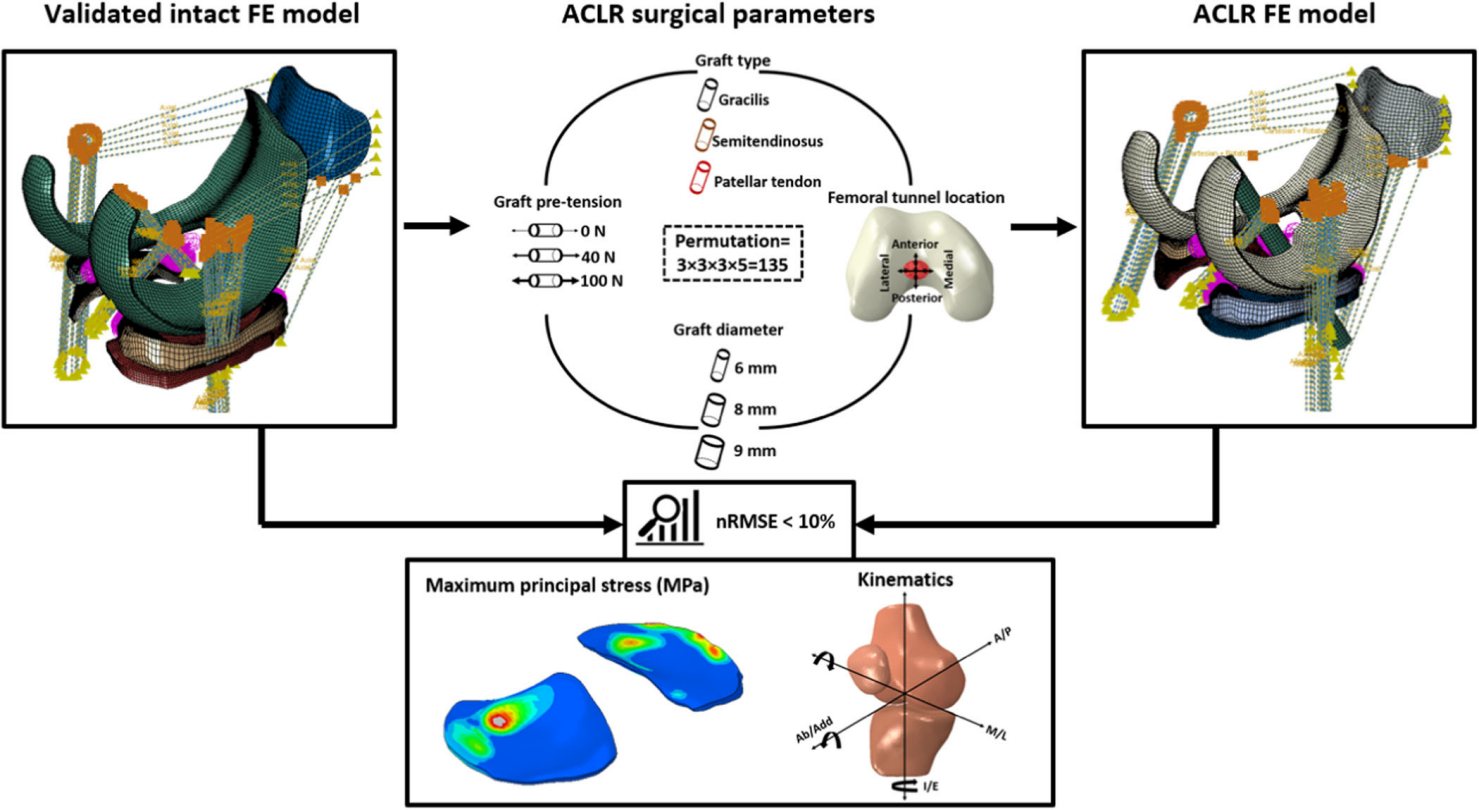
Overview of the workflow used in this study. Subject‐specific finite element models of paediatric ACLR knees were developed from a validated intact model template. The surgical parameter set was applied and tibial cartilage mechanics during the stance phase of walking simulated. Optimal surgical parameters (i.e., those closely matching intact knee biomechanics) were identified by nRMSE <10% for knee kinematics and maximum principal stresses in tibial cartilages. FE, finite element; nRMSE, normalized root‐mean‐square error.

Ethical authorization was granted by the Human Research Ethics Committee of Children's Health Queensland Hospital and Health Services (HREC/13/QRCH/197). An established NMSK‐FE modelling pipeline was used to create subject‐specific motion, loading and boundary conditions (i.e., FE model inputs) during the stance phase of walking gait for three subjects. Previously validated intact FE models of three paediatric subjects [17] served as the basis for developing subject‐specific FE models of the corresponding ACLR knees. The entire surgical parameter set (explained below) was applied to each ACLR FE model and tibial cartilage mechanics were simulated throughout the stance phase of walking. To determine effects of surgical parameters on cartilage stresses, normalized root mean square error (nRMSE) was calculated by comparing stresses in ACLR and corresponding intact model knees. Additionally, surgical parameters yielding nRMSE <10% for both knee kinematics and maximum principal stresses of medial and lateral tibial cartilages were reported for each subject as they were considered optimal (i.e., closely matching intact knee biomechanics) (Figure 1).

### Participants, medical imaging and biomechanical data acquisition

Extant data from three different typically developing children and adolescents with varying knee dimensions based on intercondylar width (i.e., small, medium and large) were selected (Table 1). Written informed consent was obtained from the legal guardians of each participant prior to commencement of any of the assessments. All experimental protocols adhered strictly to relevant guidelines and regulations, in accordance with the principles established by the Declaration of Helsinki. Each subject was provided with explicit instructions to execute a sequence of walking trials at their self‐selected pace within the Queensland Children's Motion Analysis Service (QCMAS) at the Centre for Children's Health Research (Brisbane, Australia). Marker trajectories (100 Hz, MX System; 0.017 mm accuracy relative to reference value), ground reaction forces (1000 Hz, 510 mm × 465 mm, four force plates, AMTI; with accuracy ±0.1% of applied load) and surface electromyography (EMG) signals (1000 Hz, Noraxon) were concurrently and synchronously recorded throughout the trials. The EMG signals were captured from gluteus maximus, semitendinosus, biceps femoris long head, rectus femoris, vastus medialis and lateralis, gastrocnemius, gracilis, tensor fasciae latae and sartorius. Subsequently, each participant underwent magnetic resonance imaging (MRI) of their unloaded right knee utilizing a MAGNETOM Skyra 3T scanner (Siemens).

**Table 1:** Participant characteristics used in this study.

	Small knee	Medium knee	Large knee
Gender	Female	Female	Male
Weight (kg)	49.80	65.10	67.00
Height (m)	1.50	1.67	1.79
BMI (kg/m^2^)	22.13	23.48	20.91
Lateral femoral condylar width (mm)	35.60	41.24	49.68
Medial femoral condylar width (mm)	33.46	33.26	40.83
Medial tibial plateau slope (°)	7.76	7.15	7.50
Lateral tibial plateau slope (°)	2.49	2.32	3.53
Tibiofemoral varus/valgus (°)	0.43	0.96	0.67

### NMSK‐FE modelling pipeline

In the NMSK modelling pipeline, the initial step involved modelling the external biomechanics in OpenSim [11], followed by muscle and knee contact forces using CEINMS [2, 22] (see Supporting Information, NMSK modelling pipeline).

### ACLR FE models

In this study, subject‐specific ACLR FE knee models were developed based on previously validated intact FE model using an atlas‐based approach [12, 17] (Supporting Information S1: Tables S1–S3). The ACLR FE models incorporated cylindrical graft structures with cross‐sectional diameters of 6, 8 and 9 mm, replacing the native ACL from intact FE models. Graft placement had five different locations achieved by placing different femoral tunnel apertures (guided by consultations with an experienced orthopaedic surgeon [co‐author I. A.]). Optimal graft positioning aimed to replicate native ACL footprint, and we simulated deviations up to 5 mm in medial, lateral, anterior and posterior directions (Supporting Information S1: Figure S1). The tibial tunnel was positioned to achieve anatomically appropriate placement, taking into account the native insertion site of ACL on the tibia. Specifically, our aim was to target the centre of the ACL footprint within the intercondylar area. The outside entry into the tibial tunnel was situated approximately 4 cm inferiorly from the tibial joint line and 2 cm medial to the tibial tubercle. Different graft types, representing semitendinosus, gracilis and patellar tendon, were simulated using corresponding transversely isotropic elastic materials [21] (Supporting Information S1: Table S4). Graft pre‐tension, in increments of 0, 40 and 100 N, was applied to tibial end of graft in FE models. This combination of surgical parameters and their increments resulted in 135 distinct ACLR FE models per subject (i.e., Total number of models = 3 (increments for the graft type) × 3 (increments for the graft size) × 3 (increments for the graft pre‐tension) × 5 (increments for location) = 135). Simulations were conducted using Abaqus/Standard soils consolidation solver (Daussault Systemes) for all 135 ACLR FE models per individual, and maximum principal stresses from medial and lateral tibial cartilages were reported for each FE model instance.

### Statistical analyses

The nRMSE was used to assess disparity between ACLR maximum principal stress on medial and lateral tibial cartilages and corresponding values on intact reference models. The nRMSE values were computed across 135 FE simulations per participant for both medial and lateral tibial cartilages and were presented as means with their 95% confidence intervals. To identify surgical combinations with minimal deviation from intact knee, histograms were generated for maximum principal stresses on both medial and lateral tibial cartilages and knee kinematics (i.e., anteroposterior and mediolateral translations, as well as abduction/adduction and internal/external rotations) based on nRMSE. A nRMSE < 10% was considered satisfactory, indicating minimal deviation from intact knee biomechanics [14]. A multiple linear regression analysis was conducted to determine significant independent variables, including surgical parameters (i.e., graft type, size, location and pre‐tension), as well as knee size, for predicting the dependent variables (i.e., nRMSE of medial and lateral tibial cartilages). Stepwise selection criteria were applied (entry *p* <  0.05, removal *p* >  0.1). F statistics and p‐values were used to evaluate predictive capability, while adjusted *R* ^2^ was employed to gauge effect size. Unstandardized coefficients with confidence intervals were reported for each independent variable to elucidate their direct impact on the dependent variables. Statistical analyses were performed using SPSS software (version 27, IBM), with significance set at *p* < 0.05.

## RESULTS

### Effects of surgical parameters and knee size on maximal stresses in tibial cartilages

**Figure 2:**
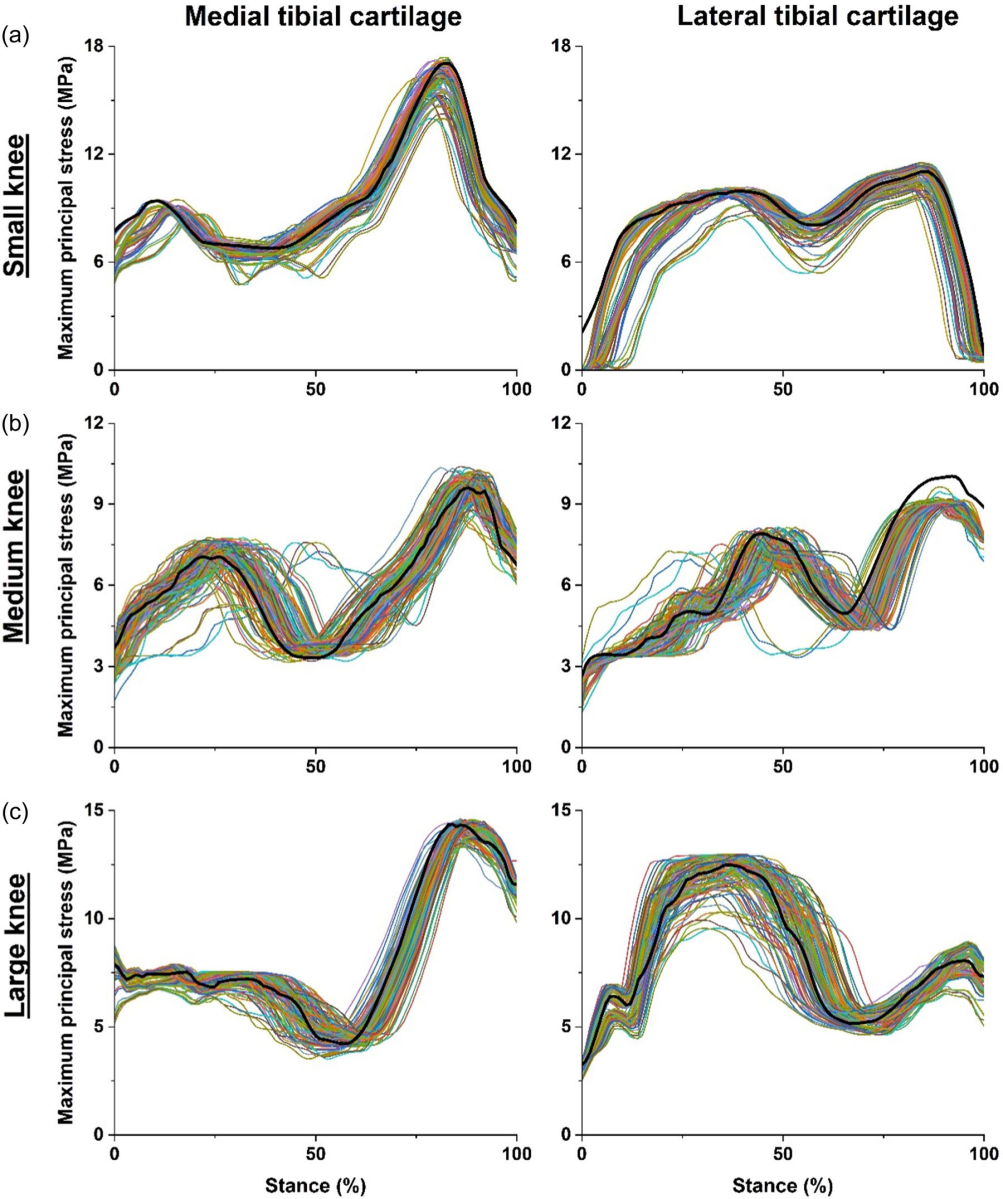
Maximum principal stresses (MPa) on medial and lateral tibial cartilages for intact (black line) and 135 ACLR finite element models (coloured lines) of (a) small, (b) medium and (c) large knees across walking stance.

Surgical parameters had substantial effects on maximum principal stresses in tibial cartilages (Figure 2). This was indicated by the many surgical configurations that resulted in maximal principal stresses in both medial and lateral tibial cartilages >10% nRMSE relative to the intact knee (Table 2). Deviation (i.e., nRMSE) in maximal stresses was greatest for both medial and lateral tibial cartilages in medium‐sized knees (i.e., 11.6 ± 5.2% and 12.1 ± 3.6%, respectively), followed by small and then large knees. Across knee sizes, deviation in maximal principal stresses was greater in lateral compared to medial tibial cartilages. Maximum principal stresses exhibited distinct patterns in magnitude and distribution across small, medium, and large knees (Figure 2). In small knee, majority (~60%) of surgical combinations led to smaller maximum principal stresses in both medial and lateral tibial cartilages compared to the intact knee (Figure 2a). In medium knees, the surgical parameter set produced divergent effects, with instances of both increased and decreased stresses on medial tibial cartilage compared to the intact knee. Notably, lateral tibial cartilage stresses in the medium‐sized ACLR knee were predominantly lower in magnitude compared to the corresponding intact knee (Figure 2b). In contrast to effects of ACLR surgery in the medium‐sized knee, ACLR in the large‐sized knee resulted in lower maximum principal stress on medial tibial cartilage compared to the intact knee, however, stresses fluctuated across the gait cycle with periods of increased and decreased magnitudes in lateral tibial cartilage compared to the intact knee (Figure 2c). As no similar computational models have been performed on the paediatric ACLR knee, a post hoc analysis was performed using G*Power software (version 3.1) [15]. The responses of the 135 simulations were used, averaged across the different knees studied, and it was found the study was powered to 95% given an *α* of 0.05.

**Table 2:** Maximum principal stresses (MPa) in medial and lateral tibial cartilage across walking stance.

	Maximum principal stresses (MPa), mean nRMSE 95% confidence interval [lower, upper bound]		
	Small knee	Medium knee	Large knee
Medial tibial cartilage	7.4 [6.7–8.1]	11.6 [10.7–12.5]	7.2 [6.7–7.7]
Lateral tibial cartilage	11.3 [10.2–12.4]	12.1 [11.5–12.7]	9.9 [9–10.8]

*Note*: Values reported as mean and 95% confidence interval of nRMSE for small, medium and large knees.

Abbreviation: nRMSE, normalized root‐mean‐square error.

A significant regression model was observed for the nRMSE of the lateral tibial cartilage (*F* = 43.776, *p* < 0.001, adjusted *R* ^2^ = 0.096) with graft pre‐tension (*p* < 0.001) emerging as a significant predictor. Each 1 N increase in pre‐tension was associated with a 2.081% decrease in nRMSE (*p* < 0.001). Additionally, deviation from optimal graft positioning led to a 0.52% increase in nRMSE (*p* < 0.004), while the increase in knee size resulted in a 0.14% increase in nRMSE (*p* < 0.024). Conversely, for the nRMSE of the medial tibial cartilage (*F* = 21.609, *p* < 0.001, adjusted *R* ^2^ = 0.049), graft pre‐tension was the sole significant predictor, with a 1.31% decrease in nRMSE observed for every 1 N increase in pre‐tension (*p* < 0.001) (Table 3).

**Table 3:** Summary of statistics for regression models and independent variables.

		Unstandardized Coefficients^a^		
Dependent variables	Independent variables	*B* (CI)	SE	*p* Value
nRMSE of lateral tibial cartilage	(Constant)	81.2 (53.8, 108.6)	14.0	<0.001
	Graft_pretension	−2.1 (−2.7, −1.5)	0.3	<0.001
	Graft_location	0.5 (0.2, 0.9)	0.2	<0.004
	Knee_size	−0.1 (−0.3, −0.2)	0.06	<0.024
nRMSE of medial tibial cartilage	(Constant)	62.5 (39.8, 85.3)	11.6	<0.001
	Graft_pretension	−1.3 (−1.9, −0.8)	0.3	<0.001

Abbreviations: *B*, unstandardized coefficient; CI, confidence interval; nRMSE, normalized root‐mean‐square error; SE, standard error.

a Interpreted as amount of increase in dependent variable per unit increase of dependent variable.

### Optimal surgical configurations: Cartilage stress and knee kinematics

**Figure 3:**
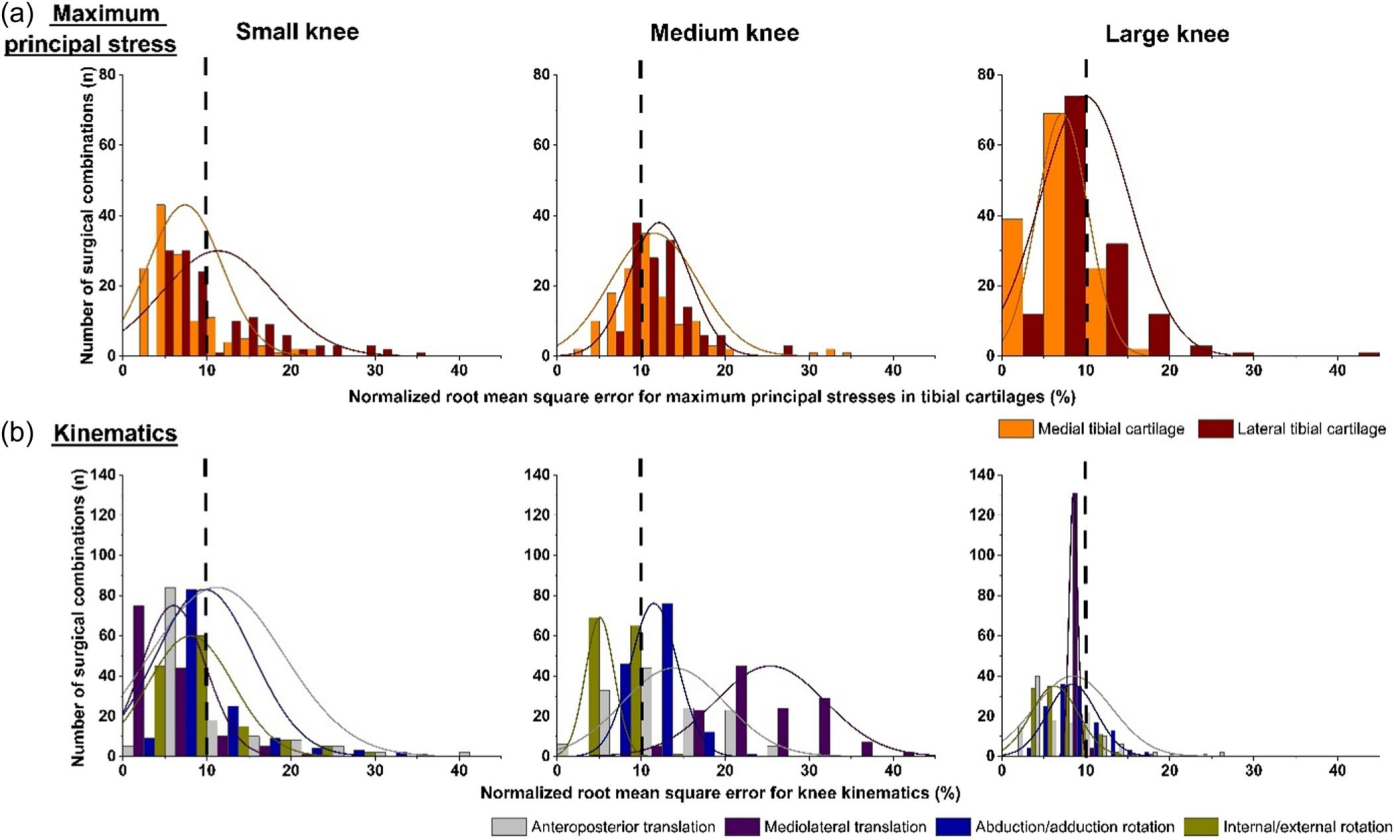
Distribution of surgical solutions, where *y*‐axis holds number of surgical combinations, *n*, from set (135) and *x*‐axis holds normalized root mean square error (%) relative to corresponding intact knee for (a, first row) maximum principal stresses (MPa) on medial and lateral tibial cartilages, and (b, second row) tibiofemoral kinematics including both rotations (°) and translations (mm). Columns represent small (left), medium (middle) and large (right) knees. Vertical dashed line represents 10% normalized root mean square error, with sets resulting in <10% error deemed satisfactory.

For the small knee, ~21% of surgical combinations yielded substantial deviation (nRMSE > 10%) in tibial cartilage stresses compared to the intact knee, particularly for medial tibial cartilage (Figure 3a). Similarly, ~38% of surgical combinations resulted in lateral tibial cartilage stresses deviating substantially from the intact knee. Approximately 60% of surgical combinations resulted in minimal stress deviations relative to the intact knee on both medial and lateral tibial cartilages concurrently. Similar trends were seen for knee kinematics, where ~59% of surgical combinations resulted in minimal rotational and translational deviations compared to the intact knee (Figure 3b). Notably, minimal cartilage stress deviations were found when graft pre‐tensions of 40 and 100 N were applied to graft diameters of 6, 8, and 9 mm. Of the set with minimal deviation, the most common configuration involved a 6 mm graft diameter positioned medially.

For the medium knee, ~59% and ~67% of surgical combinations resulted in substantial stress deviations for medial and lateral tibial cartilages, respectively, relative to the intact knee (Figure 3a). Only ~10% of surgical combinations concurrently resulted in stresses in both medial and lateral tibial cartilages similar to the intact knee. Moreover, none of the surgical set restored mediolateral translations, with only ~3% of combinations resulting in minimal (<10% nRMSE) deviation from intact knee for other DoF (Figure 3b). When considering both stresses and kinematics together, two surgical configurations achieved nRMSE<10% from intact knee. These configurations were graft diameter of 6 or 9 mm, semitendinosus graft type, 0 N pretension, and anterior graft positioning.

For a large knee, ~20% and ~36% of surgical combinations resulted in substantial stress deviations for medial and lateral tibial cartilages, respectively, compared to intact knee (Figure 3a). Approximately 52% of surgical combinations had nRMSE values <10% for both medial and lateral tibial cartilages concurrently compared to intact knee. Likewise, ~60% of surgical combinations successfully restored knee kinematics such that they mimicked intact knee kinematics (Figure 3b). Considering both stresses and kinematics concurrently, ~52% of surgical combinations resulted in minimal deviations from intact knee and were mainly characterized by graft diameters of 6 and 8 mm, pre‐tension values of 40 and 100 N and anterior graft positioning.

## DISCUSSION

The most significant finding of this study was the dependency of maximum principal stresses in tibial cartilages on both surgical parameters and subject‐specific features, such as knee anatomy, motion, and loading. Not all combinations of surgical parameters yielded acceptable knee biomechanics, highlighting the highly subject‐specific nature of optimality without overarching trends. This underscores the complexity of biomechanical responses to ACLR, which stem from intricate interactions between phenotype and NMSK factors unique to each individual. It is imperative to model this complexity to tailor surgical interventions effectively for each patient.

Consistent with our hypothesis, subject‐specific parameters such as knee phenotypes, motions, and loads influenced effects of surgical parameters on tibial cartilage stresses. The small knee had least biomechanical deviation (i.e., stresses and kinematics) across the tested surgical parameter set followed by large and then medium‐sized knees, indicating a non‐linear relationship between knee size and sensitivity to ACLR. Variation in biomechanical responses to ACLR in knees of different phenotype results from complex mechanical interaction between anatomy and NMSK biomechanics. Even for highly controlled tasks, there is large inter‐subject variation in muscle activation patterns, force sharing, and neuromuscular control, all of which interact with anatomical differences such as condylar width, tibial plateau slope, TFJ varus/valgus, and subject‐specific variables including mass and stature, to influence knee motion and cartilage mechanics post‐ACLR. Indeed, non‐linear complex behaviour at the knee has been reported in previous studies [24], however, our study adds novelty by specifically examining the interplay of surgical variations, knee anatomy, and motion/loading conditions in the understudied paediatric knee.

The regression analysis identified significant predictors of nRMSE for both the lateral and medial tibial cartilage. Graft pre‐tension emerged as a crucial factor affecting nRMSE in both regions, with higher pre‐tension associated with reduced nRMSE, indicating potentially favourable biomechanical outcomes post‐ACLR. Deviation from optimal graft positioning increased nRMSE in the lateral tibial cartilage, highlighting the importance of precise graft placement during surgery. Knee phenotype also influenced nRMSE, with larger knees exhibiting slightly higher values of nRMSE, underscoring the complexity of the relationship between surgical parameters and knee biomechanics. Although graft type and size were excluded from the regression analysis, sensitivity analysis suggests they directly and/or indirectly affect kinematics [10] and may subsequently alter tibial cartilage stresses. This discrepancy may be attributed to factors such as multi‐collinearity and overfitting, emphasizing the limitations of regression analysis in capturing the full complexity of these relationships.

Results indicated ACLR can result in both higher and lower magnitude cartilage stresses compared to corresponding intact knees (Table 2, Figure 2) depending on the specific surgical parameters used. The surgical combination associated with the highest nRMSE was accompanied by significantly larger maximum principal stress in both the medial and lateral tibial cartilage, as compared to the combination resulting in the lowest nRMSE (across diverse knee types). Abnormal cartilage stresses (e.g., under‐ or over‐loading), when chronic (e.g., during walking which is a most common motor task), are known precursors to degeneration and eventual OA onset [4, 20]. Previous studies have consistently highlighted elevated risk of early onset knee OA following ACLR [6, 9] and presence of abnormal contact loads in ACLR knees [25, 28]. The propensity for ACLR to disrupt normative cartilage stresses underscores the importance of patient‐specific surgical planning and graft selection to restore normal biomechanics (motion, loading, and tissue stress) to the post‐operative knee. With respect to the onset of knee OA, some studies indicate initial cartilage damage to the medial compartment post‐ACLR, a region more frequently affected by degeneration than its lateral counterpart [4, 6]. Conversely, other studies propose more instances of post‐ACLR knee OA in the lateral compartment [26, 27]. Our study identified greater deviation in stress patterns on lateral tibial cartilage across knee sizes (Table 1). These contradictory findings from literature underscore the complexity of the relationship between ACLR and knee OA onset, suggesting the potential subject specificity in the predilection for either the medial or lateral compartment to develop OA [26].

This study introduced a novel dimension of personalization to the field of ACLR, effectively bridging a gap in existing research methodologies and applied this novel approach to an understudied clinical population—paediatric ACLR. Diverging from conventional FE studies that have often applied fictive, generalized, and/or simplistic NMSK loading to FE models [3, 4, 20, 23], this study employed an established NMSK pipeline that integrated subject‐ and task‐specific motion, loading, and muscle activation patterns, and applied this regime to create personalized FE models of the ACLR knee. This integration ensured heightened levels of personalization when modelling knee joint dynamics. Furthermore, we conducted comprehensive analysis by simulating knee mechanics across the stance phase of walking gait. This temporal scope was crucial to capturing fluctuations in knee motion and loading across the distinct phases of walking—an aspect often neglected by prior investigations. Collectively, personalization, application to paediatrics, and temporal scope of current research constitute substantial advances in study and contribution to the knowledge of ACLR knee biomechanics.

This study is subject to several limitations that warrant consideration. First, four surgical parameters were analysed with predefined parameter spaces, but certain variables such as graft fixation angle, tibial tunnel location and graft length were excluded. These omitted variables could potentially influence knee kinematics and cartilage stresses. However, graft fixation angle was not included due to technical limitations in the FE implementation, which modelled fixation as ideal and non‐mobile. Tibial tunnel location, while minimally variant in surgical practice, was excluded because guiding articular geometry is typically unambiguous, and there are no significant limitations on tool posture or access during the extra‐articular approach. Second, the surgical parameter space was confined to three values per parameter to manage computational demands. Although this approach allowed for feasible analysis, it may have limited the resolution of findings. Expanding the parameter space or including additional surgical parameters could enhance the granularity of our analysis but would significantly increase computational demands to levels that are currently unfeasible. Third, bone deformation was not considered in the FE model due to its negligible effect on walking compared to soft tissues. Incorporating bone deformation would dramatically increase computational demands, particularly considering the extensive nature of our study, which involved 405 FE simulations across the stance phase of walking. Fourth, mechanical properties of knee tissues were not tailored to individual subjects but were instead sourced from literature. Although this approach provided a foundation for the sensitivity analyses, it may introduce variability in the results. Additionally, graft pre‐tensions were set to specific values of 0, 40 and 100 N, reflecting a clinically relevant range but potentially differing from the exact pre‐tension values achieved during surgical procedures. Last, despite efforts to mitigate uncertainties through rigorous validation procedures and statistical methods, the complexity of knee biomechanics and inherent limitations of FE modelling may still introduce some level of uncertainty in the results. Furthermore, the descriptive nature of the study limits the ability to establish causality, and future research endeavours should explore longitudinal or interventional designs to elucidate causal pathways further. From a clinical perspective, it has been shown patient variability presents a challenge to orthopaedic surgeons. Implementing a one‐size‐fits‐all approach, although efficient, will likely lead to elevated risk of re‐rupture and/or sub‐optimal loading for some patients. To mitigate this risk, results suggest personalized surgical parameter selection be informed by patient‐specific computational modelling. In the context of planning paediatric ACLR, orthopaedic surgeons may benefit from consulting orthopaedic engineers who can provide simulation services. These simulations can aid in predicting optimal surgical parameters, which can subsequently inform the surgical procedure. Future investigations are needed to determine whether such a personalized approach can reduce the incidence of poor outcomes in the paediatric ACLR population.

## CONCLUSION

In conclusion, graft type, size, location, and pre‐tension exerted complex effects on knee motion and tibial cartilage stresses in paediatric ACLR knees. Findings highlight the interplay between subject‐specific knee phenotype and NMSK factors, emphasizing the importance of tailored ACLR planning.

## AUTHOR CONTRIBUTIONS

Ayda Karimi Dastgerdi created the NMSK and FE models, extracted and analysed results, prepared figures and tables. Amir Esrafilian helped with the FE modelling and interpreted the results. Christopher P. Carty conceived the study, helped with the data gathering and the NMSK model development and interpreted the results. Azadeh Nasseri conceived the study, helped with the NMSK model development and interpreted the results. Martina Barzan helped with data gathering and interpreted the results. Ivan Astori, Wayne Hall and Rami K. Korhonen conceived the study and interpreted the results. David John Saxby conceived the study, helped with the NMSK model development and interpreted the results. All authors revised the manuscript for important intellectual content.

## CONFLICT OF INTEREST STATEMENT

The authors declare no conflict of interest.

## ETHICS STATEMENT

Ethical authorization was granted by the Human Research Ethics Committee of Children's Health Queensland Hospital and Health Services (HREC/13/QRCH/197). Written informed consent was obtained from the legal guardians of each participant before the commencement of any assessments. All experimental protocols adhered strictly to relevant guidelines and regulations, in accordance with the principles established by the Declaration of Helsinki.

## Supporting information

Supporting information.

## Data Availability

Data sets generated during and/or analysed during the current study are available from the corresponding author on reasonable request.
