# Supplementary material for: Surgical parameters influence paediatric knee kinematics and cartilage stresses in anterior cruciate ligament reconstruction: Navigating subject‐specific variability using neuromusculoskeletal‐finite element modelling analysis
Source: Knee Surg Sports Traumatol Arthrosc. 2024 Aug 6;33(3):817–27. doi: 10.1002/ksa.12413 (PMC11848988; doi:10.1002/ksa.12413)
Supplement: Supplementary file 1 — Supporting information. [file KSA-33-817-s001.docx]

# Finite element pipeline

Table S1: Element type and number of elements in the finite element knee template of atlas-based approach [3] used in this study.

| Part | Type of elements | Number of elements |
| --- | --- | --- |
| Femur | Linear hexahedral elements (C3D8P) | 19508 |
| Lateral tibial cartilage | Linear hexahedral elements (C3D8P) | 16356 |
| Medial tibial cartilage | Linear hexahedral elements (C3D8P) | 14736 |
| Patella cartilage | Linear hexahedral elements (C3D8P) | 3012 |
| Lateral menisci | Linear hexahedral elements (C3D8) | 7546 |
| Medial menisci | Linear hexahedral elements (C3D8) | 6314 |

Table S2: Material parameters of anterior cruciate ligament, posterior cruciate ligament, lateral collateral ligament, and medial collateral ligament used in this study. K – stiffness (kN) [1].

| Ligament | Bundle | K (kN) | Reference strain |
| --- | --- | --- | --- |
| Anterior cruciate ligament | aAC | 5 | 0.06 |
|  | pAC | 5 | 0.10 |
| Posterior cruciate ligament | aPC | 3 | -0.24 |
|  | pPC | 3 | -0.12 |
| Lateral collateral ligament | aLC | 2 | 0.038 |
|  | sLC | 2 | 0.038 |
|  | pLC | 2 | 0.08 |
| Medial collateral ligament | aMC | 1.83 | 0.04 |
|  | iMC | 1.83 | 0.04 |
|  | pMC | 1.83 | 0.057 |

Table S3: Material parameters for the knee joint cartilages and menisci used in this study [7].

| Material parameter | Cartilage | | | Menisci |
| --- | --- | --- | --- | --- |
|  | Superficial zone | Middle zone | Deep zone |  |
| $\boldsymbol{E}_{\boldsymbol{p}}$ (MPa) | 24 | 16.97 | 8.49 | 159.6 |
| $\boldsymbol{E}_{\boldsymbol{t}}$ (MPa) | 0.46 | 0.46 | 0.46 | 20 |
| $\boldsymbol{v}_{\boldsymbol{p}}$ | 0.42 | 0.42 | 0.42 | 0.3 |
| $\boldsymbol{v}_{\boldsymbol{tp}}$ | 0.06 | 0.08 | 0.12 | 0.01 |
| $\boldsymbol{G}_{\boldsymbol{t}}$ | 12 | 8.45 | 4.24 | 8 |
| $\boldsymbol{K}$ | 1 | 1 | 1 | - |
| $\boldsymbol{e}_{\boldsymbol{0}}$ | 4 | 4 | 4 | - |

$E_{p}$: in-plane Young׳s modulus, $E_{t}$: out-of-plane Young׳s modulus, $v_{p}$: in-plane Poisson׳s ratio,
$v_{tp}$: out-of-plane Poisson׳s ratio, $G_{t}$: out-of-plane shear modulus, $K$ : permeability, and $e_{0}$: void ratio.

Table S4: Material parameters for the grafts used in this study [10].

|  | $C_{1}$ | $C_{2}$ | $C_{3}$ | $C_{4}$ | $C_{5}$ | $\lambda^{*}$ | $D$ |
| --- | --- | --- | --- | --- | --- | --- | --- |
| Gracilis | 2.75 | 0.0 | 0.065 | 115.89 | 791.4 | 1.042 | 0.00484 |
| Semitendinosus | 2.75 | 0.0 | 0.065 | 115.89 | 512.73 | 1.042 | 0.00484 |
| Patellar tendon | 2.75 | 0.0 | 0.065 | 115.89 | 777.56 | 1.042 | 0.00484 |

$C_{1}$: constant of the Neo-Hookean model, $C_{3}$: scales the exponential stress, $C_{4}$: rate of collagen uncrimping, $C_{5}$: elastic modulus of the straightened collagen fibers, $\lambda^{*}$: the stretch at which collagen fibers start to be straightened; and $D$: the inverse of the bulk modulus.

# Neuromusculoskeletal-finite element modeling pipeline

Within the first phase of the NMSK modeling process, we used Rajagopal musculoskeletal (MSK) [12] model in OpenSim [2] (Version 3.3, Stanford University, CA, USA) to compute external biomechanics. The generic template model had 37 degrees of freedom (DoF) and 80 muscle-tendon units (MTU). This template model then underwent linear scaling for each participant to account for their inertial properties and segmental dimensions [5]. Following scaling, optimal fiber and slack lengths for each lower limb MTU were optimized to maintain dimensionless muscle fiber and tendon operating ranges [9]. Muscle strength was prescribed using Handsfield equations based on participant mass and height [4]. Once the MSK model was personalized, whole body motions, net joint forces/moments, and MTU kinematics (lengths, moment arms, lines of action) were determined using inverse kinematics (IK), inverse dynamics (ID), and muscle analysis tools, respectively.

During the second phase of the NMSK modeling process, we determined lower-limb muscle and knee contact forces using generalized loads (computed by inverse dynamics) and MTU kinematics, coupled with muscle activation patterns (conditioned EMG). Muscle dynamics were determined using Calibrated EMG-Informed Neuromusculoskeletal Modelling Toolbox (CEINMS) [11]. Initially, CEINMS was used in calibration mode to minimize discrepancies between model-predicted and experimentally measured joint moments. This optimization process included refining activation dynamics, maximum isometric forces, and optimal fiber/tendon slack lengths (within physiological bounds) to align model and experimental joint moments about the sagittal plane of the right limb's hip, knee, and ankle during selected walking trials [13]. Once calibrated, CEINMS operated in EMG-assisted mode, using conditioned EMG from ten lower limb muscles, MTU kinematics, and joint moments [3]. In EMG-assisted mode, conditioned EMG were tuned, and excitations were synthesized for the remaining 30 lower limb MTU, ensuring a balanced tracking of experimental EMG signals and joint moments, while minimizing overall muscle activation [8]. The resulting outputs from this NMSK modeling process, encompassing net joint moments, lower limb muscle forces, tibiofemoral contact forces, and patellofemoral contact forces, were subsequently integrated as loading conditions in the FE models.

In the FE modelling phase of the linked NMSK-FE approach, motions, loads, and constraints were applied to the FE knee models. To apply loads to FE models, two designated reference points were established, one for femur and one for patella, aligning with origin of knee joint coordinate system in MSK models. Nodes at interfaces between femoral cartilage and subchondral bone, as well as between patellar cartilage and subchondral bone, were coupled to femoral and patellar reference points, respectively [3, 6]. Subject-specific kinematics and kinetics from NMSK simulations of walking were applied to femoral and patellar reference points for each participant. Notably, muscle lines of action were excluded from this FE model. Thus, to maintain consistency between NMSK and FE models, tibiofemoral joint (TFJ) flexion angle (from IK analysis), knee internal/external and varus/valgus moments (from ID and respective muscle-generated moments), and tibiofemoral contact forces were applied to femoral reference point. Similarly, flexion/extension, abduction/adduction, and internal/external moments from quadriceps, along with patellofemoral contact forces, were applied to patellar reference point [3, 6]. In total, femur had five active DoF, encompassing three translations (anteroposterior, mediolateral, proximal-distal) and two rotations (abduction/adduction, internal/external). Patella had six active DoF, including three translations (anteroposterior, mediolateral, proximal-distal) and three rotations (abduction/adduction, internal/external, flexion/extension).


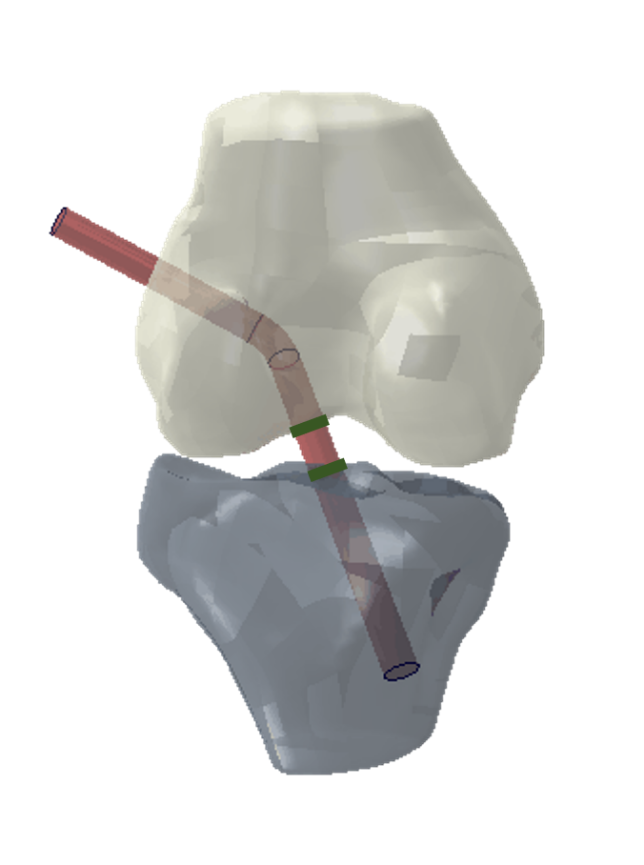


Figure S1: Optimal graft placement (highlighted in red) preserving the footprints (highlighted in green) of the native ACL.

# References

1. Blankevoort L, Kuiper J, Huiskes R, Grootenboer H (1991) Articular contact in a three-dimensional model of the knee. J Biomech 24:1019-1031.

2. Delp SL, Anderson FC, Arnold AS, Loan P, Habib A, John CT, et al. (2007) OpenSim: open-source software to create and analyze dynamic simulations of movement. IEEE Trans Biomed Eng 54:1940-1950. <https://doi.org:10.1109/tbme.2007.901024>

3. Esrafilian A, Stenroth L, Mononen ME, Vartiainen P, Tanska P, Karjalainen PA, et al. (2022) An EMG-assisted muscle-force driven finite element analysis pipeline to investigate joint-and tissue-level mechanical responses in functional activities: Towards a rapid assessment toolbox. IEEE Trans Biomed Eng 69:2860-2871. <https://doi.org:10.1109/tbme.2022.3156018>

4. Handsfield GG, Meyer CH, Hart JM, Abel MF, Blemker SS (2014) Relationships of 35 lower limb muscles to height and body mass quantified using MRI. J Biomech 47:631-638.

5. Kainz H, Hoang HX, Stockton C, Boyd RR, Lloyd DG, Carty CP (2017) Accuracy and reliability of marker-based approaches to scale the pelvis, thigh, and shank segments in musculoskeletal models. J Appl Biomech 33:354-360.

6. Karimi Dastgerdi A, Esrafilian A, Carty CP, Nasseri A, Yahyaiee Bavil A, Barzan M, et al. (2023) Validation and evaluation of subject-specific finite element models of the pediatric knee. Sci Rep 13:18328. <https://doi.org:10.1038/s41598-023-45408-5>

7. Klets O, Mononen ME, Tanska P, Nieminen MT, Korhonen RK, Saarakkala S (2016) Comparison of different material models of articular cartilage in 3D computational modeling of the knee: Data from the Osteoarthritis Initiative (OAI). J Biomech 49:3891-3900.

8. Meinders E, Pizzolato C, Gonçalves BA, Lloyd DG, Saxby DJ, Diamond LE (2022) Electromyography measurements of the deep hip muscles do not improve estimates of hip contact force. J Biomech 141:111220.

9. Modenese L, Ceseracciu E, Reggiani M, Lloyd DG (2016) Estimation of musculotendon parameters for scaled and subject specific musculoskeletal models using an optimization technique. J Biomech 49:141-148.

10. Pena E, Martinez M, Calvo B, Palanca D, Doblaré M (2005) A finite element simulation of the effect of graft stiffness and graft tensioning in ACL reconstruction. Clin Biomech 20:636-644. <https://doi.org:10.1016/j.clinbiomech.2004.07.014>

11. Pizzolato C, Lloyd DG, Sartori M, Ceseracciu E, Besier TF, Fregly BJ, et al. (2015) CEINMS: A toolbox to investigate the influence of different neural control solutions on the prediction of muscle excitation and joint moments during dynamic motor tasks. J Biomech 48:3929-3936. <https://doi.org:10.1016/j.jbiomech.2015.09.021>

12. Rajagopal A, Dembia CL, DeMers MS, Delp DD, Hicks JL, Delp SL (2016) Full-body musculoskeletal model for muscle-driven simulation of human gait. IEEE transactions on biomedical engineering 63:2068-2079.

13. Sartori M, Farina D, Lloyd DG (2014) Hybrid neuromusculoskeletal modeling to best track joint moments using a balance between muscle excitations derived from electromyograms and optimization. J Biomech 47:3613-3621. <https://doi.org:10.1016/j.jbiomech.2014.10.009>
